# The Impact of Navya-Nyāya on Mādhva Vedānta: Vyāsatīrtha and the Problem of Empty Terms

**DOI:** 10.1007/s10781-020-09453-y

**Published:** 2020-10-17

**Authors:** Michael Thomas Williams

**Affiliations:** Institute for the Cultural and Intellectual History of Asia, Austrian Academy of Sciences, 11-13 Hollandstraße, 1020 Vienna, Austria

**Keywords:** Indian philosophy, Mādhva Vedānta, Vyāsatīrtha, Empty terms, Inference, Logic

## Abstract

In this article, I explore the encounter of the Mādhva philosopher Vyāsatīrtha with the works of the Navya-Naiyāyika Gaṅgeśa Upādhyāya. The article is based on original translations of passages from Vyāsatīrtha’s *Nyāyāmr̥ta* and *Tarkatāṇḍava*. Philosophically, the article focuses on the issue of empty-terms/nonexistent entities, particularly in the context of the theory of inference. I begin by outlining the origin of the Mādhva and Nyāya positions about these issues in their respective analyses of perceptual illusion. I then contrast the role of Gaṅgeśa’s thought in the *Nyāyāmr̥ta* and *Tarkatāṇḍava* and show how Vyāsatīrtha responds to Gaṅgeśa’s ideas in different ways in those texts. I conclude with a discussion of how Vyāsatīrtha defends the Mādhva theory of “substrate-free” qualities against Gaṅgeśa in order to show that we can think and make valid inferences about nonexistent things.

## Introduction

One of the major philosophical schools in South India to come under the influence of Navya-Nyāya ideas in the early modern period (*ca*. 1500–1800) was the Mādhva tradition of Vedānta. The tradition has its origins in the thirteenth century in western Karnataka among a community of Brahmins who worshipped the Vedic god Viṣṇu-Nārāyaṇa as the supreme being. It was founded when a philosopher and religious reformer known as Madhva or Ānandatīrtha (1238–1317)^1^ rebelled against the monistic philosophy (Advaita Vedānta) prevalent in his community and formulated a system of theistic realism derived from the foundational texts of Vedic religion. Philosophers who followed Madhva (the “Mādhvas” or “Dvaita-Vedāntins”), particularly Vyāsatīrtha (1460–1539) and Satyanāthatīrtha (*fl*. 1660), wrote some of the most detailed critiques of Navya-Nyāya thought in the history of Indian philosophy.

Towards the turn of the sixteenth century, Vyāsatīrtha encountered the works of the philosopher Gaṅgeśa Upādhyāya (*fl*. 1325), who is usually regarded by modern scholars as the first Navya-Naiyāyika. Vyāsatīrtha was the first member of the Mādhva tradition to engage in detail with the ideas of Gaṅgeśa and his followers in Mithila.^2^ Williams (2014, pp. 132–133) has argued that Vyāsatīrtha was probably the first intellectual in South India to leave behind a detailed critique of Gaṅgeśa’s ideas, although there is some evidence that Gaṅgeśa’s philosophy was already being widely discussed among philosophers in South India. Vyāsatīrtha and later Mādhva philosophers were deeply influenced by their encounter with the Navya-Naiyāyikas, and Nyāya ideas led them to rethink many central issues in their theory of knowledge. The Mādhva critique of Navya-Nyāya touched on all the major issues of epistemology and logic in Indian philosophy. In this article I will focus on a single philosophical subject: the problems arising from the treatment of judgments involving “empty terms”.

As is well known, problems having to do with empty terms were at the heart of some of the most influential work in analytic philosophy at the turn of the twentieth century. The philosopher Alexius Meinong argued that every denoting phrase must refer to a thing which is, in some sense, part of reality. Fictional entities such as “golden mountains” must have at least some sort of being in order to serve as truth-makers for judgments about them. In his article *On Denoting*, Bertrand Russell famously argued that descriptive phrases have a logical form very different from the one that their grammatical form might suggest. By analysing descriptive phrases as collections of logical quantifiers and propositional functions, Russell believed he had solved many philosophical problems associated with empty terms. Russell’s work inspired new philosophical approaches to the relationship between language and reality and, to many, a new way of doing philosophy altogether.

The issue of empty terms also featured heavily in some of the central debates between philosophers in India. Indian philosophers discussed extensively the nature of terms like “hare’s horn”, “sky-flower” (*khapuṣpa*) and “the son of a barren woman” (*vandhyāsuta*). There is perhaps no one Sanskrit word used in these discussions that directly translates the expression “empty term”. However, particularly in the context of discussing inference, Indian philosophers referred to these as “unestablished”/“unexampled” (*aprasiddha*) terms. The problems that troubled medieval Indian philosophers on this subject ran along similar lines to those that concerned philosophers like Meinong and Russell. How are we able to make meaningful judgments involving empty terms? Can negative-existential judgments that seem to be about such terms (e.g. “The golden mountain does not exist”) be true/false, and, if so, how? Can formal definitions include empty terms, and can legitimate inferences contain them?

These problems drove a sizable part of the philosophical discussions that involved both the Mādhvas and the Navya-Naiyāyikas in the medieval and early modern periods. Nyāya-Vaiśeṣika philosophers had long sought to exclude empty/unestablished terms from their definitions and formal inferences. B. K. Matilal has observed that the Nyāya approach to empty terms is closely connected with their treatment of perceptual error. Indian philosophers often treated the illusion where someone mistakes a lustrous piece of mother-of-pearl for silver as a paradigmatic case of perceptual illusion. The Naiyāyikas argued for an explanation of this illusion known as the “misattribution theory” (*anyathākhyātivāda*). According to this theory, the illusion entails the misattribution of the natural kind “silverness” to the mother-of-pearl, which in reality lacks this property. It is crucial to the Nyāya theory that the different components of this illusion (the silverness and the mother-of-pearl) are parts of the real world which are mistakenly compounded together. Nyāya philosophers applied this approach to the broader problem of empty terms. Like the fake piece of silver, the “hare’s horn” is simply a fictitious compound of two unrelated parts of reality. Matilal compared this tendency to “parse-away” empty terms to Russell’s analytical position.^3^

The Mādhvas too were drawn to problems about empty terms in the context of discussing perceptual error. Mādhva philosophers were embroiled in an often acrimonious philosophical dispute with anti-realist philosophers of the Advaita school of Vedānta. According to the Advaitins, the silver/mother-of-pearl illusion simply resists our attempts to explain it. We cannot conclude that the “silver” exists, because then we cannot account for the fact that our cognition of it is later shown to be false/“sublated” (*bādhita*). Then again, we cannot conclude that it is completely *nonexistent*, like the “hare’s horn”, since in that case it would be impossible to explain how we could have had a vivid, perception-like experience of it. Advaitin philosophers argued that the “silver” is therefore simply indeterminable (*anirvacanīya*).^4^

Madhva attempted to get out of this dilemma by arguing that the “silver” *is*, in fact, “nonexistent” (*asat*). Both Jayatīrtha and Vyāsatīrtha took this to mean that the silver is completely nonexistent, like the hare’s horn or the son of a barren woman. In response to the Advaitins, they argued that we can, in some sense, have cognitions that are of nonexistent objects. Jayatīrtha and Vyāsatīrtha both accept, moreover, that we can make meaningful statements about nonexistent things, and that these statements can be truth-apt. They also accept that we can make successful inferences that involve empty terms. They theorise that this is possible because reality contains “location-free” (*anāśrita*) properties. Statements like, “The son of a barren woman is mute”, or, “The hare’s horn does not exist”, are true because nonexistent things can somehow have certain negative properties, including nonexistence itself.

Vyāsatīrtha critiqued both the Advaita and Navya-Nyāya positions about empty terms and perceptual illusions when defending this Mādhva theory in his works. His encounter with Navya-Nyāya was not simply a matter of critique, however. Mādhva philosophers from Vyāsatīrtha onward learned a great deal from Navya-Nyāya and applied the ideas and technical terms introduced by Gaṅgeśa and his followers to their philosophical disputes with other traditions. Despite their disagreements, the Mādhvas and the Navya-Naiyāyikas shared a great deal of common philosophical ground. They were both theistic traditions who were committed to realism about the empirical world. Both traditions placed a strong emphasis on sense-perception in their theories of knowledge. Both found a common enemy in the Advaitins. When debating primarily with the Advaitins in his *Nyāyāmr̥ta*, Vyāsatīrtha applied his knowledge of Gaṅgeśa’s arguments to “update” the critique of Advaita philosophy found in the works of Madhva and Jayatīrtha. On many occasions in the *Nyāyāmr̥ta*, Vyāsatīrtha adopted Navya-Nyāya conventions and positions even though they conflicted with his own, Mādhva, views.^5^

However, I wish to argue here against a way of thinking about the influence that Navya-Nyāya exerted over traditions like the Mādhvas which is taken for granted in certain discussions. This is the idea that Navya-Nyāya primarily influenced other intellectual traditions by giving them a new, rigorous terminology, a more precise and analytical way of formulating old philosophical problems. Scholars like Karl Potter, who were influenced by analytic philosophy, often seem to lean towards this position when describing the influence of Navya-Nyāya over other intellectual traditions.^6^ The available Sanskrit literature does not suggest that Mādhva philosophers regarded their encounter with Navya-Nyāya primarily as a matter of embracing an updated technical language. The Mādhvas certainly used the various technical terms that are now regarded as typical of Navya-Nyāya prose (*avacchedaka*, *avacchinna*, *nirūpaka* etc.), but Mādhva philosophers displayed a detailed engagement with a broad range of Navya-Nyāya theories, concepts and conventions, particularly the theory of inference and formal debate found in the second book of Gaṅgeśa’s *Tattvacintāmaṇi*.

Williams (2014) has examined how Vyāsatīrtha engaged with Gaṅgeśa’s new theory of pervasion (*vyāpti*) and philosophical method. In this article, I will build on these observations and explore how Mādhva philosophers learned from and critiqued Navya-Nyāya philosophy in the context of philosophical debates about empty terms. After briefly reviewing the historical context to Vyāsatīrtha’s engagement with Navya-Nyāya, I will give the background to the Mādhva/Navya-Nyāya dispute about empty terms in the works of Madhva’s leading commentator, Jayatīrtha. I will then analyse how Vyāsatīrtha applies certain Navya-Nyāya theories about “universal-negative” (*kevalavyatirekin*) inferences to his critique of Advaita philosophy in the *Nyāyāmr̥ta*. Finally, I will discuss Vyāsatīrtha’s critique of some Navya-Nyāya positions on empty terms in the *Tarkatāṇḍava.* Gaṅgeśa and his followers excluded empty terms from inferences through the concept of an inferential flaw known as “non-establishment of [the putative reason’s] locus” (*āśrayāsiddhi*). Vyāsatīrtha’s discussion of this technical point leads him into philosophically interesting debates about the nature of empty terms. It is here that he gives his clearest explanation of how location-free properties can be used to explain how we can make true judgments about nonexistent objects.

## Vyāsatīrtha and Mādhva Philosophy in the Sixteenth Century

Vyāsatīrtha’s encounter with Navya-Nyāya has its origins in a period of intense intellectual and political change for the Mādhva tradition. The sixteenth and seventeenth centuries witnessed Madhva’s followers emerge from relative isolation to become a major presence in the intellectual life of South India. This intellectual efflorescence was matched by a sudden rise of influence in key political arenas, firstly in the Hindu Vijayanagara Empire, and later in the entities that emerged as independent political powers after its demise. This success was due to a large extent to the intellectual work and political leadership of Vyāsatīrtha.

A detailed picture of Vyāsatīrtha’s life and influence at the Vijayanagara court has recently emerged from the work of Stoker (2016), who has drawn together the evidence from Vyāsatīrtha’s philosophical writings with epigraphical and biographical sources in Sanskrit. Vyāsatīrtha emerged to lead the Mādhva tradition during a period of political transformation in South India. He seems to have been dispatched to the Empire by one of his teachers just after the collapse of the Saṅgama dynasty and the ascent of the Sāḷuva lineage.^7^ He quickly forged strong links with the Empire’s rulers and became a leading figure at the temple complex at Tirupati in modern day Andhra Pradesh.^8^ He seems to have enjoyed a close relationship with the emperor Kr̥ṣṇadevarāya.^9^

The period after Vyāsatīrtha witnessed an increase in the production of philosophical literature in the Mādhva school. The Mādhva philosophers Vijayīndratīrtha (*fl*. 1560) and Vādirājatīrtha (*fl*. 1530) built on Vyāsatīrtha’s arguments in polemical tracts aimed specifically at the Advaitins. Vyāsatīrtha’s openness to other schools of philosophy led later Mādhvas to engage in a deeper way with other disciplines, including particularly Pūrva-Mīmāṃsā and grammatical science. The sixteenth century also witnessed a rise in the mystical poetry written by the Haridāsas^10^ in the Kannaḍa vernacular, including numerous works by Vyāsatīrtha and some of his followers. It was during this period that the first major replies were written to Mādhva works by other traditions such as the Advaitins who had hitherto ignored the Mādhvas.^11^ Vyāsatīrtha also engaged with Viśiṣṭādvaita philosophy extensively in his works, and Vijayīndratīrtha and his students continued the work of debating with Viśiṣṭādvaitin philosophers in Tamil Nadu.

Vyāsatīrtha was the first philosopher in his school to engage systematically with the ideas of Gaṅgeśa and his followers in Mithila.^12^ Two of his works especially display the influence of distinctively Navya-Nyāya ideas: the “Nectar of Reasoning” (*Nyāyāmr̥ta*) and the “Death-Dance of Logic” (*Tarkatāṇḍava*). The *Tarkatāṇḍava* must have been written after the *Nyāyāmr̥ta* since it refers to the *Nyāyāmr̥ta* explicitly.^13^ I would suggest that another of Vyāsatīrtha’s works, the “Resuscitation of Difference” (*Bhedojjīvana*), which is primarily a critique of Advaita Vedānta, was influenced by the work of the Mithila-based Navya-Naiyāyika Śaṅkara Miśra (*fl*. 1430), who wrote a work with a similar title, format and subject-matter. Vyāsatīrtha’s earlier commentaries on the works of Madhva and Jayatīrtha show a detailed knowledge of contemporary Nyāya ideas, but apparently no awareness of Gaṅgeśa’s work specifically.^14^

Some of the most detailed debates involving Navya-Nyāya in the Mādhva tradition unfolded in the large body of commentarial literature which grew from the *Nyāyāmr̥ta*. Leading commentaries were written on the text by Vyāsa Rāmācārya (1550–1620) and Ānanda Bhaṭṭāraka (1530–1600). The families of both of these philosophers seem to have originated in the town of Pumtamba in modern day Maharashtra.^15^ The Mādhva commentaries were clearly based on a deep engagement with the ideas of Gaṅgeśa and his commentators. The *Nyāyāmr̥ta* also seems to have inspired Advaitin philosophers to engage deeply with Navya-Nyāya ideas. The Advaitins Madhusūdana Sarasvatī (*fl*. 1570), Balabhadra (*fl*. 1610) and Gauḍa Brahmānanda (*fl*. 1700) wrote lengthy critiques of Vyāsatīrtha’s arguments, rethinking the philosophy of the medieval Advaitins in the light of Vyāsatīrtha’s critique and the theories of Navya-Nyāya.

Vyāsatīrtha’s *Tarkatāṇḍava* is, by contrast, primarily a critique of Gaṅgeśa’s ideas, as well as the arguments of some of his leading followers in Mithila. The *Tarkatāṇḍava* did not precipitate the kind of debate that the *Nyāyāmr̥ta* did. Mādhva philosophers wrote scores of commentaries on the *Nyāyāmr̥ta*, but they devoted considerably less attention to the *Tarkatāṇḍava*. This seems to have been largely because the Navya-Naiyāyikas did not reciprocate the enthusiasm with which the Mādhvas engaged with their tradition. Despite traditions suggesting that leading Navya-Naiyāyikas were impressed with Vyāsatīrtha’s learning, no reply from the Navya-Naiyāyikas is known to modern scholarship.^16^

Nevertheless, the *Tarkatāṇḍava* did attract learned commentaries from some leading Mādhva intellectuals. These philosophers lived and worked mainly in the Thanjavur area of Tamil Nadu, which became the cultural capital of what remained of the Vijayanagara Empire after the fall of Vijayanagara itself in 1565. Vijayīndratīrtha wrote a commentary on the *Tarkatāṇḍava*. Vijayīndratīrtha’s own student, Sudhīndratīrtha (*fl*. 1600), wrote a further commentary on the text. The only commentary on the *Tarkatāṇḍava* currently in print was written by Rāghavendratīrtha (*fl*. 1640). Satyanāthatīrtha (*fl*. 1670), most of whose works are only available in manuscript form, wrote a sequel to the *Tarkatāṇḍava* known as the *Abhinavatāṇḍava*. The text has been edited by scholars belonging to the Uttaradi Math in Bengaluru. What we know of Satyanātha’s life and oeuvre suggests that he was a self-consciously original thinker who sought to engage with state-of-the-art Nyāya philosophers including Raghunātha Śiromaṇi.^17^

Why did Vyāsatīrtha seek to incorporate Gaṅgeśa’s arguments into the *Nyāyāmr̥ta*, and why did he write such a detailed critique of Gaṅgeśa’s philosophy? There is some evidence that Gaṅgeśa’s arguments were already being studied in South India when Vyāsatīrtha was writing.^18^ However, the Naiyāyikas were not a major competitor of the Mādhvas in the South. While Navya-Nyāya philosophy was undoubtedly studied in South India during the early modern period, the centre of Navya-Nyāya learning clearly lay in North India in Mithila when Vyāsatīrtha was alive. The Mādhvas’ leading competitors in the Vijayanagara Empire were the Advaita and Viśiṣṭādvaita Vedāntins, and, later, the Śivādvaita and Vīraśaiva movements.^19^

This being so, what motivated Vyāsatīrtha to engage with Navya-Nyāya ideas? Until the time of Vyāsatīrtha, the Mādhvas had largely been ignored by the other traditions of Indian philosophy. Vyāsatīrtha’s three major works reflect an ambition to raise the profile of the Mādhvas as a philosophical school and to engage with other traditions of thought. The evidence from the *Tarkatāṇḍava* suggests that Vyāsatīrtha wanted to engage with cutting-edge Navya-Nyāya philosophers, including intellectuals like Jayadeva Pakṣadhara and Yajñapati Upādhyāya, who may have been senior contemporaries of his. By engaging with Navya-Nyāya, a prestigious new philosophical school which already seems to have had some standing among south Indian intellectuals, Vyāsatīrtha intended to raise the profile of the Mādhva tradition and to demonstrate that the Mādhvas should be regarded as a leading intellectual presence in the philosophical world.

Vyāsatīrtha’s engagement with Navya-Nyāya might further be taken to reflect an ambition to engage specifically with philosophers farther North in a sort of pan-Indian dispute. So far as the commentarial literature surrounding the *Nyāyāmr̥ta* was concerned, Vyāsatīrtha was certainly successful. The leading critical replies to the *Nyāyāmr̥ta* came from Bengali Advaitins based in Varanasi like Madhusūdana and Brahmānanda, both of whom seem to have been trained in Navya-Nyāya at Navadvipa in Bengal.^20^ Besides Vyāsa Rāmācārya and Ānanda Bhaṭṭāraka, whose families originated in Maharashtra, Mādhva philosophers from North India like Vanamālī Miśra (*fl*. 1680), who seems to have come from Bihar,^21^ in turn wrote replies to the critiques of Advaitin philosophers.

Vyāsatīrtha’s work on Navya-Nyāya further seems to be part of a broader move towards an “interdisciplinary” approach which can already be discerned in the works of the fourteenth-century Mādhva philosopher Viṣṇudāsācārya. In the early modern period, Advaitin philosophers like Appayya Dīkṣita (*fl*. 1585) seized upon the fact that Madhva himself had grounded his philosophical ideas in controversial “lost” texts, which many modern scholars now doubt really existed.^22^ Vyāsatīrtha’s engagement with key specialist disciplines of Indian thought (grammatical science, Mīmāṃsā and Navya-Nyāya in particular) reflect this project to “normalise” Mādhva philosophy and to confer mainstream respectability on it by justifying it in the terms of these established sciences. These factors no doubt ultimately contributed to the success of Vyāsatīrtha’s work in attracting replies from leading scholars outside of the Mādhva tradition.

## The Background to Vyāsatīrtha’s Philosophy: Nonexistence in Madhva and Jayatīrtha

Matilal argued that the Nyāya treatment of empty terms is closely connected with the Naiyāyikas’ theory of perceptual error. While discussing the Nyāya treatment of perceptual illusions, he likens the Nyāya “misjudgment” theory (*anyathākhyātivāda*) to Russell’s approach to empty terms:

The theory [of *anyathākhyāti*] … is generalized in Nyāya to explain other philosophical problems connected with vacuous names and descriptions which are apparently meaningful, although there is nothing that they name or that answers such descriptions. This is also a relevant analysis in connection with what may be called the old Russell-Meinong controversy over the problem of fictional entities. Part of the philosophic insight that might have prompted Russell to propound his theory of definite description can be seen to be at work as the Nyāya tackles the problem of empty terms in logic by generalizing the ‘misplacement’ (*anyathākhyāti*) theory. For certain problems of perception can be transposed back into the problems of reference. For example, if I cannot *see* a nonexistent object, how can I name it, or try to refer to it or describe it? Moreover, the initial name-giving occasion, as the modern (Kripke’s) theory of reference would emphasize, requires a ‘perceptual’ sort of situation (comparable to baptism).^23^

It was in the context of debating perceptual error that Madhva and Jayatīrtha began to formulate theories about empty terms and fictional objects. Whereas the ancient Naiyāyikas were writing primarily against the different schools of Buddhism, the Mādhvas’ main rivals were the Advaita Vedāntins. Again, the Advaitins argue that the “silver” we might mistake a piece of mother-of-pearl for is “indeterminable”/“indeterminate” (*anirvacanīya*) from the ontological point of view. It clearly cannot be a real part of the objective situation that gives rise to the illusion, because then we could not explain the “sublating” cognition (*bādhakajñāna*) which subsequently informs us that the “silver” is not really there. Then again, the silver cannot be *entirely nonexistent* either, for then we could not perceive it at all. This treatment of perceptual illusions evolved primarily in the works of tenth century Advaitin philosophers like Prakāśātman and Vimuktātman, who sought to ground their larger metaphysical views about the empirical world in the analysis of such illusions.^24^

The realist schools of Indian philosophy, including the Mādhvas and the Naiyāyikas, argued, by contrast, that perceptual illusions are actually mundane events which are perfectly compatible with realism about the empirical world. Jayatīrtha based his theory of illusion on the Nyāya *anyathākhyāti* theory, according to which the illusion happens because we misattribute a real natural kind (silverness) to an individual (the mother-of-pearl) which actually lacks it. Jayatīrtha identifies his theory of perceptual error as a version of the Nyāya approach with a twist. Madhva had countered the Advaitins’ argument for indeterminacy by arguing that we *can*, in fact, cognise nonexistent things. Jayatīrtha agrees with the Naiyāyikas at least insofar as he accepts that perceptual illusions must involve the active misidentification of one thing with another. Unlike the Naiyāyikas, however, Jayatīrtha accepts that the object of our false perception (the “snake”, the “silver” etc.) is *simply nonexistent.* Nevertheless, according to Jayatīrtha real individuals can serve as “prototypes” for the entity we seem to perceive in illusions.^25^ In other words, the “silver” portion of the cognition can be traced back to some part of reality (a real piece of silver in a shop, for instance), but this prototype cannot legitimately be said to be the erroneous cognition’s *object*. Unlike in the Nyāya theory, the *particular piece* of silver that appears in the erroneous cognition does not have any direct correlate in the real world.

There are legitimate questions about how accurately Jayatīrtha represents the Nyāya theory here, and the true extent of his difference with them.^26^ Nevertheless, these differences of approach translated into different ideas about how to treat empty terms. Madhva’s arguments with the Advaitins ultimately led his followers to adopt a distinctive approach to the problem of nonexistence. According to Jayatīrtha and Vyāsatīrtha, we can think and reason about nonexistent entities because nonexistent things can, somehow, have certain qualities.

In his commentary on one of Madhva’s critiques of Advaita philosophy, the *Tattvoddyota*, Jayatīrtha outlines the essentials of the Mādhva approach to nonexistence when debating the doctrine of indeterminacy with a hypothetical Advaitin opponent. Indian philosophers accepted that our introspective intuitions about episodes of perceptual illusion are useful and need to be accounted for when formulating theories. Both the Mādhvas and the Advaitins accept that perceptual illusions involve a “sublating” or cancelling cognition (*bādhakajñāna*), which corrects the initial erroneous cognition, e.g., “This ‘silver’ is nothing but a piece of mother-of-pearl!”. After this cognition, the victim of the illusion potentially has an introspective intuition along the lines of, “‘False/Illusory/Unreal’ silver appeared [to me]” (*mithyaiva rajataṃ pratyabhāt*). The debate is about the precise meaning of the term *mithyā* here. According to the Advaitins, the word *mithyā* means “indeterminate”. In the relevant passage, Jayatīrtha argues that *mithyā* simply means “nonexistent”:

*Objection (Advaitin)*: … If the word *mithyā* does not refer to what is indeterminate, then [you] must specify what it means. *Reply (Mādhva)*: True enough! We say [that it means] “nonexistent”.

*Objection (Advaitin)*: In that case it follows that [the word *mithyā*] is meaningless! For [one] cannot [say] “What is nonexistent exists” (*asad asti*), because [that is] contradictory. And if [the word *mithyā*] is meaningless, then it cannot be a word at all. *Reply (Mādhva)*: Wrong! Because there is nonexistence in the form of “being the counterpositive of a constant absence”. For, the statement “[It is] *mithyā*” does not mean “[It is] a hare’s horn” or so on. For then [one] would not [say] “The hare’s horn is *mithyā*”. So what [does it mean]? “It does not exist.” And so the [sublating judgment] “The silver is in fact *mithyā*” (*mithyā eva rajatam*) means “There is the constant absence of silver”.

*Objection (Advaitin)*: How can something that itself is nonexistent have the quality of being a counterpositive? *Reply (Mādhva)*: Why do you ask “how”? For, unlike [the trope] colour and so on, the state of being a counterpositive does *not* depend on the existence of its locus. For, “being a counterpositive” is nothing more than “being an object of a cognition that is conducive to a cognition of an absence”. And we shall demonstrate [later in this work] that there can be a cognition even of what does not exist.^27^

According to the Mādhvas, the “silver” is simply nonexistent (*asat*). According to the Advaitins, on the other hand, the word *mithyā* indicates that the silver is *indeterminate* from the point of view of its ontological status. When critiquing the Advaita position here, Jayatīrtha advances two major points which would become central to Vyāsatīrtha’s argumentation. The first is Jayatīrtha’s definition of “nonexistence” itself. His definition, “being the counterpositive of a constant absence”, forms the basis of Vyāsatīrtha’s own definition of the term. In the *Nyāyāmr̥ta*, for instance, Vyāsatīrtha defines “existence” and “nonexistence” as follows:

Existence is said to consist in *not* being the counter-positive of an omni-spatio-temporal absence; something that is superimposed and something that is totally nonexistent *are* the counterpositives [of such an omni-spatio-temporal absence].^28^

Each entity, in other words, has a “location-range”, a set of locations in which it is present. This range is extended temporally as well as spatially. According to Vyāsatīrtha, something is nonexistent if it has a null location range. Something is nonexistent, in other words, if it is absent from all locations at all points in time. To be “existent”, on the other hand, is simply to have the absence of nonexistence so-defined: something exists if it has a *non*-*null* location range. In other words, something exists if it is present in just one location at a single point in time. These are, of course, fully contradictory qualities: nothing can have a null *and* a non-null location range, and everything must have one of the two.

In this passage, Jayatīrtha says a second thing which is particularly pertinent to our discussion. He here touches on a theory which would become central for Vyāsatīrtha in his refutation of Gaṅgeśa in the *Tarkatāṇḍava*. This is the theory of “substrate-free” (*anāśrita*) qualities, that is, qualities that lack an existent locus. In order for the “silver” to be absent, it must be the counterpositive (*pratiyogin*) of some absence. However, this means that it must at least have the relational abstract counterpositiveness (*pratiyogitva*). But how can something that does not exist have properties of any sort? Jayatīrtha’s solution to this problem is to argue that, while certain qualities (for instance, tropes [*guṇa*s] like colour, taste and so on) can only be present in *existent* entities, other qualities, including counterpositiveness, *do not* need to occur in an existent substrate. Vyāsatīrtha will argue against Gaṅgeśa and the Navya-Naiyāyikas that this is the only theory that can satisfactorily reconcile a realist attitude towards thought and language with the facts of empty terms.

## Applying Gaṅgeśa in the *Nyāyāmr̥ta*

Much of Vyāsatīrtha’s work on Navya-Nyāya drew on the detailed defence of Nyāya inferential theory that Gaṅgeśa had given in the second book of the *Tattvacintāmaṇi*. The Naiyāyikas sought to preclude unestablished terms from their definitions and formal inferences. According to the Naiyāyikas, a standard inference seeks to prove that some quality (the probandum, *sādhya*) is present through some relationship in a certain location (the subject, *pakṣa*) because some other quality (the reason, *hetu*) is also present there. The standard example is the case where we infer that there is fire on a mountain because there is smoke on the same mountain. We are able to make this inference because we know that the probandum (fire) “pervades”/is invariably concomitant with the reason (smoke). According to the Naiyāyikas, we simply cannot make inferences where the subject is an unestablished term (e.g., “The son of a barren woman cannot speak …”) or where the probandum itself is unestablished (e.g., “The earth has a golden mountain on it …”).

This restriction on inference led Navya-Naiyāyikas like Gaṅgeśa into certain problems regarding a type of inference which they referred to as “universal-negative” (*kevalavyatirekin*) inference. Much of Vyāsatīrtha’s critique of the Advaitins’ theory of indeterminacy in the *Nyāyāmr̥ta* draws on a problem which arises from Gaṅgeśa’s discussion of this issue. The standard example of a universal-negative inference used by Gaṅgeśa is based on the classical Vaiśeṣika theory of substance (*dravya*). It effectively captures the process of defining the substance “earth” (*pr̥thivī*):

Earth is different from the other [substances and categories], because [it has] earthness (*pr̥thivī itarabhinnā, pr̥thivītvād*).

In its full form, the inference would read:

*Thesis:* Earth is different from the other substances and categories.

*Reason*: Because it has earthness.

*Example*: That which is *not* differentiated from these other substances and categories does *not* have earthness, as in the case of water.

*Application*: And earth does not *not* have earthness.

*Conclusion*: Therefore, earth is not *not* differentiated from the other substances and categories.

By double negation, it follows that earth *is* different from the other substances and categories. From now on I will refer to this inference simply as the “earth-inference”.

What exactly does it mean to say that earth is “different from everything else”? Apart from Raghunātha Śiromaṇi and certain thinkers who were influenced by him, Navya-Nyāya philosophers accepted the view that reality ultimately consists of seven categories: substance (*dravya*), trope (*guṇa*), motion (*karma*), natural kind (*jāti*), ultimate particulariser (*viśeṣa*), the inherence relator (*samavāya*) and absence (*abhāva*). In order to show that earth has the property of being “different from everything else”, we need to show first of all that earth is different from the categories apart from substance. (As Vyāsatīrtha’s commentator, Rāmācārya, points out, Vyāsatīrtha, following Gaṅgeśa, does not include the category of absence on the list when discussing the earth-inference.)

Earth is a substance, however, and it must thus also be differentiated from all the remaining substances. According to classical Vaiśeṣika theory there are eight substances besides earth: water (*ap*), fire (*tejas*), air (*vāyu*), ether (*ākāśa*), time (*kāla*), space (*diś*), self (*ātman*) and the internal faculty/“mind” (*manas*). The earth-inference thus tries to establish that earth is different from the five categories other than substance/absence, along with the eight other substances. The probandum can therefore be analysed as a complex property, which consists of thirteen “mutual absences” (*anyonyābhāva*) or differences from the following things:

**Table:** 

Substances	Categories
1. Water	9. Trope
2. Fire	10. Motion
3. Wind	11. Natural kind
4. Ether	12. Ultimate differentiator
5. Time	13. Inherence
6. Space	
7. Self	
8. Mind	

In the *Tattvacintāmaṇi*, Gaṅgeśa defined universal-negative inference as follows:

A universal-negative [reason] is one which has no homologue (*sapakṣa*), where the pervasion is grasped through negative concomitance.^29^

A *sapakṣa*, a “similar instance” or “homologue”, is a location other than the subject where the probandum is known to be present by the person making the inference. If there is no homologue, then how can we grasp the pervasion that is essential to the inferential process? The *Nyāyasiddhāntamuktāvalī* says that in the universal-negative mode of inference the *absence* of the reason pervades the *absence* of the probandum.^30^

To help clarify this, we can use modern logic. “Positive pervasion” (e.g., “Where there is smoke, there is fire”), understood from the Naiyāyikas’ point of view, can be translated into the formula:

$$\left( {\forall {\text{x}}} \right) \, ({\text{Hx}} \to {\text{Sx}}),$$

where the predicates H and S correlate with the reason and probandum respectively. Universal-negative pervasion is the contraposition of this, that is:

$$\left( {\forall {\text{x}}} \right) \, (\sim {\text{Sx}} \to \sim {\text{Hx}}),$$

i.e. “the *absence* of the reason pervades the *absence* of the probandum”.

Why is universal-negative inference problematic? In essence, the problem that drives much of Vyāsatīrtha’s arguments in the *Nyāyāmr̥ta* has to do with unestablished terms. As we saw above, the Naiyāyikas attempted to bar such dubious entities from entering into inferences at all. However, the probandum in the earth-inference must be a *unique* property. Obviously, nothing other than earth in the Vaiśeṣika universe can have the property of being different from all of the things that earth is different from! In the *Tattvacintāmaṇi*, Gaṅgeśa assesses the problems surrounding this kind of inference as follows:

*Objection*: In the universal-negative [inference] “Earth is different from the others [= the remaining substances and categories], because [it has] earthness”, the probandum is unestablished. Hence, there cannot be (1) the determination of the negative-concomitance [of the probandum and the reason], nor (2) subjectness, nor (3) the cognition of that [= the subject] qualified by the probandum produced by the reason. For, all of those are produced by the cognition of the probandum.

Or, let us assume that the probandum *is* established. In that case, if the reason *is* found where [the probandum] is established, then the reason has a *positive* concomitance [with the probandum]; if [it is] *not* found [where the probandum is found], then [the reason] is “uncommon”.^31^

The underlying problem is that universal-negative inference by its very nature seems to be incompatible with the requirement that the probandum in any inference must not be an unestablished term. A universal-negative inference has the requirement (a) that the number of known locations in which there is the absence of the probandum and the presence of the reason is zero, i.e. that there are no known instances in which the probandum is absent and the reason is present. However, it also has the extra requirement (b) that the person making the inference is aware of no *positive* concomitance between the probandum and the reason. Nevertheless, there is a further requirement, applicable to all types of inference, (c) that the probandum must be an established (*prasiddha*) property.

Requirement (c) implies that the person making the inference must be aware that the probandum is present in one other location than the subject of the inference at hand (i.e. in a “homologue”). Since a location must either be subject to the presence of any quality or its absence, one of two things follows from this. Either the reason is present in the homologue or it is absent from it. If it is *absent*, then there is a deviation (*vyabhicāra*) and the inferential cognition cannot arise. More formally, this means that what is taken to be a reason in such an inference suffers from the defect of “being ‘uncommon’”: (*asādhāraṇya*); that is, it is absent from something that possesses the probandum. On the other hand, if the reason is *present* in the location where the probandum is known to be present, then it follows that the reason is not of the universal-negative sort, but of the *anvayavyatirekin* variety; that is, it has both a positive *and* negative concomitance with the probandum.^32^

This is precisely the problem that drives much of Vyāsatīrtha’s critique of certain inferences made by Advaitin philosophers in the *Nyāyāmr̥ta*. Towards the beginning of the text, Vyāsatīrtha states several key inferences found in the works of philosophers like Ānandabodha and Citsukha. These Advaitins opposed the realism of other Brahmanical schools like Nyāya, Vaiśeṣika and Pūrva-Mīmāṃsā, and held that the world of our senses is simply an illusion mistakenly superimposed on *brahman*. In the *Nyāyāmr̥ta*, Vyāsatīrtha considers among other arguments the following three inferences to prove this anti-realist position:

The subject of [our] dispute [= the empirical world] is illusory, because [it has] perceptibility, insentience and finitude, just like the “silver” [mistakenly superimposed on] mother-of-pearl (*vimatam mithyā, dr̥śyatvāt, jaḍatvāt, paricchinnatvāt; śuktirūpyavat*).^33^

Towards the beginning of the *Nyāyāmr̥ta*, Vyāsatīrtha analyses each part of these inferences in turn. He first considers the probandum, “illusoriness/unreality” (*mithyātva*), which can mean “indeterminacy” (*anirvacanīyatā*) according to the Advaitins. Vyāsatīrtha’s critique of indeterminacy here draws on many of Gaṅgeśa’s ideas about universal-negative inference. Indeterminacy is the claim that the empirical world and the perceptual illusions that prefigure it are indeterminate from the point of view of their ontological status. Like the object we seem to see in perceptual illusions, the empirical world cannot be truly existent, for then it would be identical with ultimate reality, that is, *brahman,* and it would not be liable to sublation. Then again, it cannot be *completely nonexistent*, since we could not then explain how we are able to perceive and interact with it. One way of explaining indeterminacy, therefore, is to say that the world lacks both the qualities of “existence” and “nonexistence”.

In the *Nyāyāmr̥ta*, Vyāsatīrtha presents three ways that the Advaitin philosopher might further analyse indeterminacy. He asks his Advaitin opponent:

It is said—You yourself have defined “illusoriness/unreality” (*mithyātva*) in five different ways by refuting another viewpoint. Of those, in regard to the first [= indeterminacy], do [you] mean:

D^1^: the absence of a compound entity (*viśiṣṭa*), namely “nonexistence qualified by existence”?

D^2^: a pair of properties: the constant absence of existence and the constant absence of nonexistence?

D^3^: a compound entity, namely “the state of having the constant absence of nonexistence qualified by the state of having the constant absence of existence”?^34^

In his critique of these arguments, Vyāsatīrtha draws together Madhva’s own critique of the Advaitin’s position with the Navya-Nyāya theory of inference. Madhva himself argued that the Advaitins’ inferences suffer from both the flaw of “[the subject’s] having-an-unestablished-qualifier/probandum” (*aprasiddhaviśeṣaṇatā*) and “proving what has already been proved” (*siddhasādhana*).^35^ As Vyāsatīrtha uses the term here, the flaw of *siddhasādhana* amounts to proving something that one’s opponent already accepts.

In the case of D^2^, Vyāsatīrtha concurs with Madhva that there is “partial” redundancy of proof: the Advaitin is attempting to prove that the world has the constant absence of nonexistence, something that the Mādhvas, who accept that the empirical world exists, take to be an established fact. However, in the *Nyāyāmr̥ta*, Vyāsatīrtha reconsiders Madhva’s arguments in the light of the example of the earth-inference:

Let it be that somehow, as in the case of the thirteen mutual absences in [the probandum of the inference]: “Earth is different from the others [= the remaining substances and categories] because [it has] earthness”, in [the inference at hand] the constant absences of existence and nonexistence are established separately, and hence the flaw of having-an-unestablished-qualifier (*aprasiddhaviśeṣaṇatā*) does not apply. Nevertheless, [D^2^ fails because] there is, further, the flaw of establishing what has already been established (*siddhasādhana*) as far as the constant absence of nonexistence*—*which constitutes one part [of the probandum*—*is concerned. For, what is established does not become unestablished by virtue of being articulated in connection with what is unestablished.^36^

In the [thesis of the earth-inference]*—*“Earth is different from the others [= the remaining substances and categories]”*—*, by contrast, the individual mutual absences from water and so on are *not* established in what is connected with earthness. And [D^2^ also fails] because the example lacks the probandum. The reason, earthness, on the other hand, is a universal-negative one. The negative-determination of the probandum, i.e. the thirteen mutual absences, is possible purely because the thirteen mutual absences, though they have different substrates, are grasped in a single collective cognition.^37^

Vyāsatīrtha here uses the case of the earth-inference as a sort of “precedent” by which to judge the Advaitin’s inferences. In the *Tattvacintāmaṇi*, Gaṅgeśa proposed several solutions to the conundrum posed by universal-negative inference, but Vyāsatīrtha draws mainly on one of these solutions when responding to the Advaitins. According to Gaṅgeśa, the probandum in the earth-inference can be said to be established before the inference takes place. For, even though the person making the inference might have never encountered all thirteen absences existing in *one and the same location*, the thirteen individual absences that make up the probandum could be established *separately* in the other parts of the Vaiśeṣika universe before the inference takes place. For instance, the mutual absence from water could be established in fire, the mutual absence from fire in wind, and so on.^38^

Like the probandum in the earth-inference, the probandum in the Advaitin’s inference defined as D^2^ is a “partite” probandum: it consists of two separate absences—the constant absence of existence and the constant absence of nonexistence. These absences might, so Vyāsatīrtha reasons, be established *separately* before the inference brings them together in a single collective cognition. Vyāsatīrtha’s commentator Śrīnivāsatīrtha points out that the constant absence of nonexistence would be established in what is existent and, vice versa, the constant absence of existence would be established in what is nonexistent.

Vyāsatīrtha here seems to agree with the line of reasoning that Gaṅgeśa defends in the *Tattvacintāmaṇi*. However, Madhva and Jayatīrtha denied that universal-negative pervasion can form the basis of inference *directly* in the way that Gaṅgeśa and the Naiyāyikas suggest.^39^ This commitment leads Vyāsatīrtha to refute the very arguments that he seems to accept in the *Nyāyāmr̥ta* in the *Tarkatāṇḍava*. This shows that Vyāsatīrtha took a different approach towards Gaṅgeśa and Navya-Nyāya in the two texts. In connection with his use of the “statement of disagreement” (*vipratipatti*) at the beginning of the *Nyāyāmr̥ta*, for instance, Vyāsatīrtha shows a willingness to work with Nyāya theories of debate and inference, even if they are not strictly compatible with Mādhva theories.^40^

This sample of Vyāsatīrtha’s critique of Advaita in the *Nyāyāmr̥ta* is typical for the way he uses Navya-Nyāya ideas in the earlier parts of that text. The substance of Vyāsatīrtha’s critique of the Advaitins’ inferences is already found in the works of Madhva and Jayatīrtha. However, Vyāsatīrtha takes Madhva’s case and reconsiders it in the light of an engagement with the inferential theory of the *Tattvacintāmaṇi*. This engagement is not solely a matter of rephrasing older arguments about metaphysics using a more refined technical language. Vyāsatīrtha certainly makes abundant use of the signature technical terms associated with Navya-Nyāya in this section of the *Nyāyāmr̥ta*, primarily the use of the term “limiter” (*avacchedaka*) in connection with the relational abstracts involved in inference. However, he also shows a deep engagement with the particular theories and arguments surrounding the nature of universal-negative inference which we find in the *Tattvacintāmaṇi*.

## Empty Terms and Nonexistence in the *Tarkatāṇḍava*

The Mādhvas and the Navya-Naiyāyikas shared a great deal in common philosophically. Like the Naiyāyikas, the Mādhvas believe that the world is a determinate totality of mind-independent objects. They concur with the Naiyāyikas that we apprehend those objects directly, not through the mediation of mental images, sense-data or the like. Both traditions agree that truth involves some type of correspondence relationship between mental judgments and things in the world. Like the Naiyāyikas, the Mādhvas lean towards a sort of empiricistic philosophy of knowledge: perception is the primary means of knowledge and, accordingly, it enjoys epistemological priority over inference and verbal testimony.

However, despite their shared philosophical outlook, the Mādhvas differed from the Naiyāyikas on many issues in the theory of knowledge. Madhva himself developed an epistemological theory which is, in many respects, at odds with the Nyāya system. The *Tarkatāṇḍava* contains Vyāsatīrtha’s defence of Madhva’s theories against the new ideas of Gaṅgeśa and fifteenth-century Navya-Naiyāyikas from Mithila including Jayadeva Pakṣadhara, Yajñapati Upādhyāya and Pragalbha Miśra. As I have observed in my discussion of Vyāsatīrtha’s definition of pervasion (*vyāpti*), Vyāsatīrtha’s critical encounter with Gaṅgeśa and his followers was a philosophically productive one. It led him to reexamine and, in many cases, substantially rethink Madhva and Jayatīrtha’s theories about knowledge. Vyāsatīrtha nevertheless drew on the rich intellectual resources found in the works of his tradition’s leading thinkers to show that Mādhva solutions to the problems of knowledge are ultimately superior to the Naiyāyikas’.

The *Tarkatāṇḍava* sees Vyāsatīrtha return to the issue of nonexistence, although this time primarily as a critique of the Navya-Nyāya position. The Navya-Naiyāyikas held that we cannot make inferences that seem to be about empty/unestablished terms like “the son of a barren woman” or “the golden mountain”. Put more technically, the requirement is that the inferential subject (*pakṣa*) is not such an empty term. Many Navya-Naiyāyikas expressed this limitation on inference through an inferential flaw known as *āśrayāsiddhi*.^41^ A favourite example of such an inference, which Vyāsatīrtha uses frequently in the *Tarkatāṇḍava,* is:

The son of a barren woman cannot speak, because [it] lacks sentience (*vandhyāsuto na vaktā, acetanatvāt*).

Vyāsatīrtha gives an extensive critique of the idea that *āśrayāsiddhi* is a separate type of inferential flaw in the *Tarkatāṇḍava*. Neither Madhva nor Jayatīrtha accepted that *āśrayāsiddhi* is a separate flaw and Vyāsatīrtha argues that there are only two types of inferential flaws pertaining to the reason.^42^ However, his arguments draw him into a philosophically rich discussion about the nature of fictional entities and the limits of reason. Vyāsatīrtha’s approach to empty terms is ultimately conditioned by the works of Madhva and Jayatīrtha and their debate about perceptual illusions with the Advaita Vedāntins. Vyāsatīrtha’s final conclusion is that the Navya-Naiyāyika has no choice but to admit, like him, that we *can* think, speak and reason about nonexistent entities. He believes that the fact that we can do so is only explicable if we accept that nonexistent things can have certain qualities, or that certain qualities, including nonexistence itself, are “substrate-free” (*anāśrita*). Unlike positive qualities such as colour, existence and so on, these “location-free” qualities need not occur in an existent locus.

Why do the Naiyāyikas believe that we cannot make inferences that involve empty terms? As a realist, Vyāsatīrtha, like the Naiyāyikas, shares the assumption that mental judgments are true or false depending on whether they correspond to some external situation. Like the Navya-Naiyāyikas, Vyāsatīrtha tends to work within the assumption that judgments about an entity (the *dharmin*) typically locate some quality (*dharma*) within it. On the face of it, we seem to be able to make truth-apt judgments about nonexistent entities, for instance, that “The golden mountain is nonexistent”; or, perhaps, “The son of a barren woman cannot speak”.

In the inference, “The son of a barren woman cannot speak, because [it] lacks sentience”, a *negative* property (“non-speakerness”, *avaktr̥tva*) is seemingly ascribed to a nonexistent entity, namely the “son of a barren woman”. Why, in the view of the Navya-Naiyāyika, does this inference fail? After dismissing several alternative definitions of *āśrayāsiddhi*, Vyāsatīrtha gives voice to the following Nyāya argument in support of the Naiyāyikas’ position that this inference is invalid:

However, [the flaw of *āśrayāsiddhi*] is “having a nonexistent substrate” (*asadāśrayatva*), as in the inference, “The son of a barren woman cannot speak, because [it has] insentience”. Now, is that a flaw because: (1) since what is nonexistent cannot have qualities, (a) [it] cannot be the substrate of the quality that is to be proved by the inference and hence there would be [the inferential flaw of] “contradiction” (*bādha*) [and] (b) since there would be the absence of the reason [in the subject], there would be [the inferential flaw of] “non-establishment” (*asiddhi*); or, (2) since [a nonexistent entity] cannot be the object of [*any*] judgment, it cannot be involved in *any* linguistic act, be it an ascription *or* a denial; or, (3) since [what is nonexistent is not] amenable to the valid means of knowledge, it cannot be an object of those means of knowledge; or, (4) because, if it were *not* a flaw, then it would follow that other things [which clearly cannot be valid inferences would have to be regarded as such]; or, (5) because two qualities that lack any locus cannot be related by pervasion, that is, the relationship of invariant collocation.^43^

According to the Navya-Nyāya model of inference, an inference must prove that some property is present in a certain subject because some other property is present in the same subject. This being so, the Naiyāyika can argue that any inference that seeks to prove something about a nonexistent subject would suffer from the flaws pertaining to the reason that are known as “contradiction” (*bādha*) and “non-establishment [of the reason]” (*asiddhi*). Technically, the reason in an attempted inference is said to be “contradicted” if the subject it is taken to be present in is known by some other means to have the *absence* of its probandum. A reason is said to be “unestablished”, on the other hand, if it is not present in the inferential subject at all.^44^

According to the Naiyāyika’s argument as Vyāsatīrtha presents it here, both of these flaws apply to the inference in question because something nonexistent like the “son of a barren woman” cannot have qualities at all. Accordingly, the “son of a barren woman” cannot have the quality of “not being a speaker” (*avaktr̥tva*), and so we know that it must have the absence of the probandum. The reason is therefore a pseudo-reason of the “contradicted” variety. For the same reason, the subject cannot have the reason attributed to it by the inference, i.e. “lacking sentience”. Hence, the reason cannot be established to exist in the subject, and it could be said to be a pseudo-reason of the “unestablished” variety. So the inference cannot be valid.

In the passage translated above, Vyāsatīrtha goes on to voice several other reasons that the Naiyāyikas could give to show that we cannot make inferences about nonexistent things. They might argue that we cannot make inferences about nonexistent things because we simply cannot truly think about such things at all. How could we ascribe/deny properties to/of “the son of a barren woman” if we cannot become mentally acquainted with it in the first place? Similarly, the Naiyāyikas hold that we cannot know nonexistent things through any of the valid means of knowledge. How could we ascribe properties to something that we cannot know through at least one of the means of knowledge? The Naiyāyikas might also argue that, if we accept that the inference about the “son of a barren woman” is valid, then we would have to accept that any number of other inferences which are clearly untenable are valid. Vyāsatīrtha presumably would not want to accept the validity of inferences like, “The golden mountain is heavy, because it is made of gold”, but how are they to be ruled out if we are able to make inferences with unestablished subjects? A final reason is that the Naiyāyikas tended to assume that pervasion entails a community of locus between the probandum and the reason. A knowledge of such an invariable concomitance is essential for inference; however, how can we know that two properties share a common locus if they have no locus in the first place?

Vyāsatīrtha responds to all of these arguments systematically in the *Tarkatāṇḍava*, but I will here focus on how he responds the first charge. Again, the Naiyāyika’s argument is that the putative reason in the inference, “The son of a barren woman cannot speak, because it is insentient”, is “contradicted” and “unestablished”, because nonexistent things cannot have any kind of property. The essence of Vyāsatīrtha’s response to this claim of the Naiyāyikas is that it is self-defeating to claim that we cannot make inferences about what does not exist. For, any argument to prove that we cannot make such inferences must *itself* assert or deny things about nonexistent entities on the basis of some reason. In other words, the Nyāya case is hopelessly contradictory: it is impossible to deny that we can meaningfully speak or reason about nonexistent entities, because that denial itself requires that we think and reason about nonexistent things. Anybody who claims that nonexistent things cannot properly be the subject of an inference must *themselves* make inferences about nonexistent things in order to persuade their opponent that this is so!

Vyāsatīrtha makes his case as follows:

The first two [grounds for *āśrayāsiddhi*’s being a flaw] are untenable. For, since the probanda you have mentioned—“not being the substrate of the probandum” and “not being the substrate of the reason”—, as well as [your] reason—“the state of lacking [all] qualities”—cannot be present in what is nonexistent, you yourself are guilty of contradiction and [non-establishment of reason]. And because if the qualities you have specified *can* be present there [= in what is nonexistent], because they are negative in form, then for the very same reason can the qualities *I* accept—non-speakerness, insentience and so on—also [be present in what is nonexistent]. For I too do not accept that *positive* [qualities can be present in things that do not exist].

And if *you* are not guilty of contradiction and [non-establishment of reason] because the quality of not being the substrate of the quality to be established and [the reason] can exist even in the absence of the quality of being the locus of the absence of the quality to be established and [the reason], then *I* too am not guilty of contradiction [and having an unestablished reason], since the quality of not being the locus of speakerness can exist even in the absence of the quality of being the locus of the absence of speakerness.^45^

Once again, the Naiyāyika is claiming that the “reasons” in inferences about nonexistent things must be “contradicted” and “unestablished” because nonexistent things can have neither the probandum nor the reason that the inference ascribes to them. The basis of the Naiyāyika’s argument is that nonexistent things cannot have the reason/probandum because they cannot have properties at all. However, as Vyāsatīrtha points out, the Naiyāyika’s case to support these claims seems *itself* to involve inferences about nonexistent things. These inferences could be written in an abbreviated form as follows:

1. *Thesis*: What is nonexistent cannot be the locus of the probandum,

*Reason*: Because it lacks qualities.

2. *Thesis*: What is nonexistent cannot be the locus of the reason,

*Reason*: Because it lacks qualities.

These inferences seem to be self-confuting: it is self-contradictory to attempt to prove, through inference, that no nonexistent thing can be the subject of an inference. Indeed, even the reason in these inferences—the “state of lacking qualities” (*nirdharmakatva*)—seems to be self-contradictory, since it is *itself* a sort of quality. The pattern of argument articulated by Vyāsatīrtha here has a good pedigree in Mādhva philosophy. As we saw above, it is already found in Madhva’s critique of the Advaitins’ doctrine of indeterminacy. According to Advaitin philosophers, the object involved in perceptual illusions—the “silver” etc.—cannot be entirely nonexistent, since we have a vivid, perceptual-like cognition of it. One of Madhva’s key arguments against this position was that the claim that, “We cannot cognise what is nonexistent”, is a sort of pragmatic contradiction: the very fact that someone is able to make judgments about something that is nonexistent proves that that person can cognise it somehow.^46^

Vyāsatīrtha now proceeds to consider arguments that the Naiyāyika might give to rescue his position. One explanation is that the Naiyāyika *does* accept that nonexistent entities can have *negative* qualities. A nonexistent entity cannot be the locus of *positive* qualities such as sentience, speakerhood and so on, but it *can* possess certain negative qualities. (Vyāsatīrtha now explicitly places a limitation on the Mādhva theory: nonexistent entities can only be the locus of negative qualities like *non*existence and *in*sentience.) The second solution offered by the Naiyāyika in this passage seems to be that negative judgments can be made about something, even if it does *not* have the relevant absence. For instance, the “son of a barren woman” may not be the locus of the “absence of the quality of ‘being a speaker’” (*vaktr̥tvābhāva*), but it can still be said to have the quality of “not being the locus of speakerness” (*vaktr̥tvānāśrayatva*).

The underlying problem for the Naiyāyika is that, even if these solutions are successful, they could equally serve to vindicate Vyāsatīrtha’s position that we need to accept that there are “substrate-free” qualities if we are to explain how we seem to be able to make meaningful judgments about nonexistent things. Vyāsatīrtha believes that their underlying philosophical assumptions compel the Navya-Naiyāyikas to adopt his theory of substrate-free qualities. He argues:

For, there are various sorts of quality. Some are located in a substrate, such as colour tropes and so on. Yet others are located in one thing, while they affect something else, as cognition and so on [which are located in the self but affect] pots and so on. Some are substrate-free, like nonexistence and so on, because [we have] the uncontradicted judgment, “The hare’s horn is nonexistent”. For, otherwise, the nonexistence of [the hare’s horn] could not be established.^47^

Vyāsatīrtha here advances a philosophical theory of true judgments about empty terms along the lines that Jayatīrtha had expressed in texts such as the *Tattvoddyotaṭīkā* and the *Nyāyasudhā.* Seemingly true judgments about nonexistent entities can only be true because nonexistent entities can somehow have certain negative qualities, including nonexistence itself.

The idea that certain properties do not require an existent locus might seem to offend that “feeling for reality which ought to be preserved even in the most abstract studies”, as Russell put it.^48^ However, Vyāsatīrtha believes that a commitment to the type of realist principles that the Navya-Naiyāyikas themselves took for granted leads us inexorably to this conclusion. Navya-Nyāya metaphysicians largely assumed that reality must conform to the ways in which we speak and think about it. The goal of metaphysical analysis is to specify how reality must be in order to account for, in the most parsimonious way possible, the factual occurrence and validity of the true judgments made by human beings.^49^ Vyāsatīrtha argues that the fact that we have true (or at least uncontradicted) judgments that seem to ascribe qualities to nonexistent things compels us to accept that reality must contain qualities that lack any existent locus. The basic principles of Nyāya realism can only be explained using the Mādhva theory of substrate-free qualities.

## Conclusion

The analysis of empty terms was at the heart of much metaphysical dispute in India during the early modern period. The Mādhva treatment of empty terms has its origins in the philosophy of perception and Madhva’s critique of indeterminacy. In the *Tarkatāṇḍava*, we see Vyāsatīrtha make the case for the theory of substrate-free qualities against the Naiyāyikas. Vyāsatīrtha’s argument here seems to trade on a deep contradiction in Nyāya philosophy. On the one hand, the Navya-Naiyāyikas sought to exclude empty/unestablished terms from their inferences and definitions. Moreover, their analysis of perceptual illusion leads to an analytical approach to the problem of nonexistence which explains empty terms as fictitious combinations of parts of the real world. On the other hand, the Navya-Naiyāyikas were committed to the view that reality is somehow isomorphic with our true judgments about it, and that we can infer the structure of reality from those judgments. Vyāsatīrtha’s critique of the Nyāya position suggests that these two tendencies in Nyāya thought exist in an uneasy tension, to say the least. His conclusion is that the only way to satisfactorily reconcile a realist approach to thought and language with the facts of empty terms is to assume that certain qualities like “nonexistence” need not occur in an existent locus.

Aside from the philosophical implications of this discussion, these passages from the *Nyāyāmr̥ta* and *Tarkatāṇḍava* are representative of Vyāsatīrtha’s encounter with Navya-Nyāya in general. In the *Nyāyāmr̥ta*, he seems to be prepared to accept aspects of Gaṅgeśa’s theory of inference that he sets out to refute in the *Tarkatāṇḍava*. As the passage considered from the *Nyāyāmr̥ta* shows, Vyāsatīrtha based his arguments on a detailed engagement with Gaṅgeśa’s theory of inference, not simply the application of new, more precise technical terms. This readiness to learn from Navya-Nyāya, however, is combined with a commitment to the superiority of his own, Mādhva philosophical tradition. Vyāsatīrtha and the Mādhvas were, like the Navya-Naiyāyikas, committed to realism about the empirical world, and in the passages of the *Tarkatāṇḍava* considered, Vyāsatīrtha indicates that he shares deep assumptions about the proper method of metaphysical analysis. However, he ultimately seeks to demonstrate that only the Mādhva theory of substrate-free qualities can adequately explain the facts about empty terms.

Vyāsatīrtha’s decision to engage with the Navya-Naiyāyikas was a decisive intellectual intervention in his school’s history. His critique of Advaita philosophy attracted the attention of scholars from all over India and signaled the entrance of the Mādhvas into the mainstream philosophical world that had hitherto largely ignored them. Later Mādhva thinkers such as Satyanāthatīrtha displayed an ambition, like Vyāsatīrtha, to engage with cutting-edge Nyāya philosophers, and to emphasise the originality and “newness” of their own work. Vyāsatīrtha’s critique of Navya-Nyāya in the *Tarkatāṇḍava* does not seem to have met with an enthusiastic response from the Navya-Naiyāyikas themselves, but it did inspire much critical philosophical thought in his own tradition.

Scholarship by Ganeri (2011) has emphasised the philosophical importance of the critical work on metaphysics undertaken by Navya-Nyāya philosophers in Navadvipa and Varanasi in the early modern period. Until recently, there was comparatively little interest in the works of Vyāsatīrtha and his critique of Gaṅgeśa’s *Tattvacintāmaṇi*. However, in the last few years there has been a long-overdue surge in interest among scholars in Vyāsatīrtha’s contributions to Indian philosophy. Hopefully this growing focus on the “second founder” of the Mādhva tradition will continue to shed new light on how he and his followers engaged with Navya-Nyāya philosophy in the early modern period.

## Footnotes

1 All dates for Mādhva philosophers in this article are drawn from Sharma (1961). The dates of other philosophers are based on the online version of Volume 1 of Karl Potter's *Encyclopedia of Indian Philosophies: Bibliography:* http://faculty.washington.edu/kpotter/ (accessed June 3, 2020).

2 Sharma (1961, p. 252) has claimed that Jayatīrtha was aware of Gaṅgeśa and that he cites a verse from the *Tattvacintāmaṇi* in his *Nyāyasudhā*. I have not been able to locate this verse in any modern edition of the text. Sharma claims that Jayatīrtha was active as an intellectual mainly in the later part of the fourteenth century. This suggests that it is at least possible that Jayatīrtha was aware of Gaṅgeśa’s work, assuming that Gaṅgeśa himself flourished in the earlier part of the fourteenth century. Even if Jayatīrtha did become aware of Gaṅgeśa’s writings at some point in his life, Gaṅgeśa’s work seems to have had little impact on his own.

3 Matilal (1986, pp. 201–213).

4 For an extensive discussion of the concept of indeterminacy in the works of the tenth-century Advaitin Vimuktātman, see Schmücker (2001).

5 See Williams (2014).

6 See for instance Potter (2011, p. 86).

7 See *Vyāsayogicarita*, p. lxxxiii.

8 See Stoker (2016, pp. 88–95) for a discussion of Vyāsatīrtha’s relationship with Tirupati.

9 For recent discussions of Vyāsatīrtha's relationship with Kr̥ṣṇadevarāya, see Stoker (2016, pp. 17–45) and Stoker (2011).

10 The Haridāsas were a group of Mādhva-affiliated mystical poets who wrote hymns in the Kannaḍa vernacular. Vyāsatīrtha’s teacher, Śrīpādarāja (= Lakṣmīnārāyaṇatīrtha), is widely considered to be the founder of this movement. Vyāsatīrtha himself composed several hymns in Kannaḍa, and is considered to have been the preceptor of two of the most eminent Haridāsas, Purandaradāsa and Kanakadāsa.

11 See McCrea (2014) for a discussion of Vyāsatīrtha's historical treatment of Advaita philosophy in the *Nyāyāmr̥ta* and his influence over the Advaitin philosopher Appayya Dīkṣita. See Duquette (2019) for a discussion of the influence of the *Tarkatāṇḍava* over Appayya’s work.

12 For a survey of some of the Navya-Naiyāyikas whom Vyāsatīrtha alludes to in the *Tarkatāṇḍava*, see Williams (2014, p. 131).

13 See Sharma (1961, p. 306).

14 See Williams (2014, p. 132).

15 See Sharma (1961, p. 533) for the origins of these two thinkers and the expansion of the Mādhva tradition in Maharashtra.

16 See Williams (2014).

17 See Sharma (1961, pp. 445–449).

18 See Williams (2014, pp. 132–133).

19 See Stoker (2011) for an analysis of Vyāsatīrtha's critique of Viśiṣṭādvaita soteriology in the *Nyāyāmr̥ta*. See Duquette (2015, pp. 21–22) for a discussion of how the Mādhvas, beginning with Vijayīndratīrtha, responded to Appayya Dīkṣita’s work on Śivādvaita philosophy. Vijayīndratīrtha is said to have had a dispute with a Vīraśaiva guru at Kumbakonam. See Sharma (1961, p. 399).

20 See Sastri (1984, pp. 89–90) for more on the life of Gauḍa Brahmānanda. Sastri claims that he was a contemporary of Jagannātha Paṇḍitarāja and a classmate in Navadvipa of one of Raghunātha Śiromaṇi's leading commentators, Gadādhara Bhaṭṭa. See Gupta (2006) for a discussion of Madhusūdana's life and education. See also Minkowski (2011) for an overview of Advaitin intellectuals in early modern India.

21 See Sharma (1961, pp. 387–388).

22 See Mesquita (2000).

23 Matilal (1986, p. 201).

24 See Schmücker (2001) for an extensive analysis of the doctrine of indeterminacy in the works of the Advaitin philosopher Vimuktātman. The Advaitins’ theory is also discussed in the context of the Nyāya *anyathākhyāti* theory of illusion by Duquette and Ramasubrahmanian (2009).

25 For this interpretation of Jayatīrtha's theory, see Sharma (1962, p. 212).

26 See Williams (2017a) for an extended discussion of these aspects of Jayatīrtha's theory of perceptual illusion.

27 *nanu* … *anirvacanīyasya yadi na mithyāśabdo vācakas tarhi tadvācyaṃ vācyam | satyam | asad iti brūmaḥ | evaṃ tarhi nirarthaka iti prāptam | na hy asad astīti sambhavati, vyāhatatvāt | nirarthakatve ca padatvavyāghāta iti cet, maivam | atyantābhāvapratiyogitvalakṣaṇasyāsattvasya vidyamānatvāt | na hi mithyety asya śaśaviṣāṇādikam ity arthaḥ | tathā sati śaśaviṣāṇaṃ mithyeti na syāt | kiṃ nāma? tan nāstīti | tathā ca mithyaiva rajatam ity asya nāsti rajatam, rajatātyantābhāvo* ’*stīty** arthaḥ | svayam asataḥ kathaṃ pratiyogitvam iti cet, kim iha katham? na hi pratiyogitvaṃ rūpādivad dharmisattāsāpekṣam, abhāvajñānopayogijñānaviṣayatāmātrasya pratiyogitvatvāt | asato ’pi pratītim upapādayiṣyāmaḥ |* (*Tattvoddyotaṭīkā*, p. 32).

28 *trikālasarvadeśīyaniṣedhāpratiyogitā* / *sattocyate ’dhyastatucche taṃ prati pratiyoginī* // (*Nyāyāmr̥ta,* p. 249).

29 *kevalavyatirekī tv asatsapakṣaḥ, yatra vyatirekasahacāreṇa vyāptigrahaḥ* | (*Tattvacintāmaṇi*, p. 582).

30 *Nyāyasiddhāntamuktāvalī*, p. 501.

31 *nanu pr̥thivī itarebhyo bhidyate, pr̥thivītvād iti vyatirekiṇi sādhyam asiddhaṃ | tathā ca na vyatirekanirūpaṇam, na vā pakṣatvam, na vā liṅgajanyasādhyaviśiṣṭatajjñānam; teṣāṃ sādhyajñānajanyatvāt | atha sādhyaṃ prasiddham | tadā yatra prasiddham, tatra hetor avagame* ’*nvayitvam; anavagame ’sādhāraṇyam* | (*Tattvacintāmaṇi*, pp. 596–597).

32 See Williams (2013) for a discussion of this problem in Jayatīrtha's *Tattvoddyotaṭīkā*.

33 *Nyāyāmr̥ta*, p. 24.

34 *ucyate—mithyātvaṃ hi tvayaiva pakṣāntaraniṣedhena pañcadhā niruktam | tatrādye kiṃ sattve saty asattvarūpaviśiṣṭasyābhāvo ’bhipretaḥ? kiṃ vā sattvātyantābhāvāsattvātyantābhāvarūpadharmadvayam? yad vā sattvātyantābhāvavattve saty asattvātyantābhāvavattvarūpaṃ viśiṣṭam?* (*Nyāyāmr̥ta*, pp. 52–53). Vyāsatīrtha's use of the logic relating to a “compound” or “qualified” entity (*viśiṣṭa*) may have been influenced by Gaṅgeśa's chapter on subjectness (*pakṣatā*) in the *Tattvacintāmaṇi*, given that Vyāsatīrtha shows a close familiarity with this portion of Gaṅgeśa's text earlier in the *Nyāyāmr̥ta*. *Cf*. *Tattvacintāmaṇi*, pp. 431–432.

35 For instance, in his brief critique of Advaita philosophy, a work which is most widely known as the “Refutation of the Inference to Establish [that the Empirical World has] Illusoriness” (*Mithyātvānumānakhaṇḍana*), Madhva argues: *vimatam mithyā, dr̥śyatvāt; yad itthaṃ tat tathā, yathā śuktirajatam* … *anirvacanīyasyāsiddher aprasiddhaviśeṣaṇaḥ | asadvailakṣaṇye mithyātvasya siddhasādhanatā |* (*Mithyātvānumānadūṣaṇa* [= *Mithyātvānumānakhaṇḍana*], p. 58). “The thing under dispute is illusory (*mithyā*), because [it has] perceptibility; whatever is so [= perceptible] is as such [= illusory], as in the case of the silver superimposed upon shell. … Since what is indeterminable is not established, [the subject] has an unestablished qualifier. [And] illusoriness amounts to establishing what has already been established [by me, the realist] in respect of the state of being different from what is nonexistent.”

36 This is a reference to Jayatīrtha's commentary on Madhva's *Mithyātvānumānakhaṇḍana*. *Cf*. *Mithyātvānumānakhaṇḍana*, p. 36.

37 *pr̥thivī itarabhinnā, pr̥thivītvād ity atra trayodaśānyonyābhāvānām ivātrāpi sattvāsattvātyantābhāvayoḥ pratyekaṃ prasiddhatvena kathaṃ cid aprasiddhaviśeṣaṇatvābhāve ’pi, asattvātyantābhāvāṃśe siddhasādhanāc ca | na hi siddham asiddhena sahoccaritam asiddhaṃ bhavati | pr̥thivī itarabhinnety atra tu jalādyekaikānyonyābhāvo* ’*pi na pr̥thivītvopahite siddhaḥ | dr̥ṣṭāntasya sādhyavaikalyāc ca | pr̥thivītvahetus tu kevalavyatirekī | trayodaśānyonyābhāvarūpasādhyasya vyatirekanirūpaṇaṃ tu bhinnāśritānām api trayodaśānyonyābhāvānāṃ samūhālambanaikajñānopārūḍhatvamātreṇa yuktam* | (*Nyāyāmr̥ta,* p. 53). Vyāsatīrtha's commentator Śrīnivāsatīrtha writes: *nanu pr̥thivītvahetau vyatirekiṇi dr̥ṣṭāntābhāve trayodaśānyonyābhāvānāṃ sādhyabhūtānāṃ prasiddhyabhāvena sādhyavyatirekanirūpaṇaṃ na syād ity ata āha—trayodaśeti | jalāditrayodaśānyonyābhāvānāṃ tejaḥprabhr̥tiṣu pratyekaṃ pratyekaṃ jñānānantaram, ete trayodaśānyonyābhāvā iti samūhālambanarūpaikajñānopārūḍhānāṃ prasiddhisambhavena vyatirekanirūpaṇaṃ sambhavatīti bhāvaḥ* | (*Nyāyāmr̥taprakāśa*, *Nyāyāmr̥ta*, p. 65).

38 *Cf*. *atha vā jalādīnāṃ trayodaśānyonyābhāvās trayodaśasu prasiddhāḥ pr̥thivyāṃ sādhyante | ata evākāśe vyatirekiṇā jalādimilitapratiyogikānyonyābhāvāpratītāv api trayodaśānyonyābhāvāḥ sādhyā iti nānvayitvāsādhāraṇye* | (*Tattvacintāmaṇi*, p. 615). “Alternatively, the thirteen mutual absence of water [and the remaining substances and categories], being well-established in the thirteen [remaining substances and categories themselves], are established [by the earth-inference] in respect of earth. For this very reason can the thirteen mutual absences [from the remaining substances and categories] be established in the ether through a universal-negative inference, even though there is no [prior] experience of a mutual absence, the counterpositive to which is the sum of water [and the remaining substances and categories]. Hence [the inference] is *not* based on a positive correlation, and the reason is *not* a pseudo-reason of the ‘uncommon’ variety.” This passage has been translated and analysed by Phillips (2010, pp. 478–479).

39 According to Jayatīrtha in his “Manual on Knowledge/The Means of Knowledge” (*Pramāṇapaddhati*: p. 276), the Mādhva position is as follows. Only a positive pervasion is *directly* operative in producing inferential knowledge. However, in certain cases, this pervasion itself needs to be established via a further inference. Take, for instance, the inference: “All living bodies have a soul, since they have breath and so on”. Here, the subject (the aggregate of living bodies) exhausts all possible locations where we could become aware of a positive concomitance between the probandum and the reason. However, Jayatīrtha is aware that we can infer a positive pervasion (A pervades B) from its contraposition (~B pervades ~A); that is, we can infer (∀x) (Bx → Ax) from (∀x) (~Ax → ~Bx). Consequently, while negative-pervasion might not play a *direct* role in the inferential process, it can certainly support it *indirectly*, by helping us to establish the positive pervasion that really forms the basis of the inference. See Williams (2011, pp. 113–114).

40 *Cf*. *Tarkatāṇḍava,* pp. 180–183. For a discussion of Vyāsatīrtha's arguments in the *Tarkatāṇḍava*, see Williams (2011, pp. 117–128).

41 The famous Navya-Nyāya training manual, the “Pearl-Necklace of the Established Positions of Nyāya” (*Nyāyasiddhāntamuktāvalī*), defines *āśrayāsiddhi* as follows: *āśrayāsiddhiḥ pakṣe pakṣatāvacchedakasyābhāvaḥ | yatra kāñcanamayaḥ parvataḥ vahnimān iti sādhyate, tatra parvato na kāñcanamaya iti jñāne vidyamāne kāñcanamaye parvate parāmarśapratibandhaḥ phalam* | (*Nyāyasiddhāntamuktāvalī*, p. 249). “'Non-establishment of substrate' (*āśrayāsiddhi*) is the absence of the limiter of subjectness (*pakṣatāvacchedaka*) in the subject. Where [someone] undertakes to prove that, 'The golden mountain possesses fire', if there is the cognition, 'A mountain does not consist of gold', the result is the blocking of the consideration (*parāmarśa*) with regard to the golden mountain.”

42 Madhva accepted that there are three sorts of inferential flaw, pertaining to the three different elements of an inference: the thesis, the reason and the example. In the *Tarkatāṇḍava*, Vyāsatīrtha elaborates Madhva's theory as follows: *yatsadbhāve liṅgābhimataṃ pramāṃ na janayati, so* ’*numānadoṣaḥ … sa ca trividhaḥ—pratijñāhetudr̥ṣṭāntabhedena … hetoḥ sādhyapramājananasāmarthyābhāvo hetudoṣaḥ | sa dvividhaḥ—asiddhir* ^1^*avyāptiś ceti*^1^ *| samucitadeśe hetor abhāvo* ’*siddhiḥ | vyāptiviraho* ’*vyāptiḥ |* (*Tarkatāṇḍava,* pp. 220–222.) ● 1. *avyāptir iti* (ga, rā). “If, when *x* is present, what is taken to be a reason (*liṅga*) does not generate an [inferential] knowledge, then *x* is a flaw pertaining to inference. *…* And that is of three sorts, owing to the difference between thesis, reason and example. A flaw pertaining to the reason is the absence of the capacity to generate knowledge of the probandum pertaining to the reason. It is of two sorts*—*‘non-establishment’ (*asiddhi*) and ‘non-pervasion' (*avyāpti*). ‘Non-establishment' is the absence of the reason in the appropriate place. 'Non-pervasion' is what is devoid of pervasion.” All variant readings are given with the *sigla* used in the Mysore edition of the *Tarkatāṇḍava.*

43 *kiṃ tu vandhyāsuto na vaktā, acetanatvād ity ādāv ivāsadāśrayatvam | tasya tu doṣatvaṃ kim—asato nirdharmakatvena sādhyadharmānāśrayatvena bādhāt, sādhanābhāvenāsiddher vā? pratītyaviṣayatvena vidhiniṣedharūpasakalavyavahārābhājanatvād vā?* ^1^*aprāmāṇikatvena pramāṇānaṅgatvād*^1^ *vā? tasyādoṣatve ’tiprasaṅgād vā? niradhikaraṇayor dharmayor niyatasāmānādhikaraṇyarūpavyāptyabhāvād vā?* (*Tarkatāṇḍava,* pp. 240–241). ● 1. *apramāṇatvenānaṅgatvād vā* (ṭa); *aprāmāṇikatvād vā* (rā).

44 See Gupta (1980, pp. 140–143) for a discussion of the definitions of these terms in Nyāya philosophy.

45 *nādyau | asati tvaduktayoḥ sādhyasādhanadharmānāśrayatvarūpayoḥ sādhyayor nirdharmakatvarūpasya sādhanasya cāsambhavena tavāpi bādhādiprasaṅgāt | tvaduktadharmāṇām abhāvarūpatvāt tatra* ^1^*sambhave ca*^1^ *tata eva maduktāvaktr̥tvācetanatvāder api sambhavāt | bhāvarūpāṇāṃ tu mayāpy anaṅgīkārāt | yadi ca sādhya*^2^*dharmādyabhāvādyāśrayatvābhāve*^2^ *’pi sādhyadharmādyanāśrayatvasya sattvāt tava na* ^3^*bādhādiḥ*^3^*, tarhi* ^4^*vakr̥tvābhāvāśrayatvābhāve*^4^ *’pi* ^5^*vakr̥tvānāśrayatvasya*^5^ *sattvān mamāpi na bādhādi* | (*Tarkatāṇḍava,* pp. 241–242) ● 1. *sambhavena* (ka, ga); 2. *dharmābhāvādyāśrayatvābhāve* (ka, ga, ṭa, rā); 3. *bādhādi* (ga, rā, ṭa [*pc.*]); 4. *vādyāśrayatvābhāve* (ga, rā); 5. *vaktr̥tvādyanāśrayatvasya* (ga, ṭa, rā).

46 See *Viṣṇutattvanirṇaya*, pp. 21–22.

47 *vicitrā hi dharmāḥ | ke cid āśritāḥ, yathā rūpādayaḥ | ke cid anyāśritā anyoparañjakāḥ, yathā jñānādayo ghaṭādīnām | ke cid anāśritāḥ, yathāsattvādayaḥ, śaśaśr̥ṅgam asad ity abādhitapratīteḥ | anyathā tasyāsattvāsiddheḥ* | (*Tarkatāṇḍava*, p. 244). Rāghavendratīrtha comments: *keṣāṃ cid āśrayābhāve* ’*sattvam eva dr̥ṣṭam iti katham āśrayābhāve dharmāṇāṃ sattvam ity ataḥ svabhāva evāyaṃ dharmāṇām iti bhāvena sudhoktam evāha—vicitrā hīty ādinā* | (*Nyāyadīpa*, *Tarkatāṇḍava*, p. 244).

48 See Russell (1920, p. 169).

49 See Williams (2017b) for the application of this principle in the works of Raghunātha Śiromaṇi. See also Ganeri (2011) for a general discussion of realism and metaphysical analysis in Navya-Nyāya.
